# Sequential Hybrid Therapy With Pulmonary Endarterectomy and Additional Balloon Pulmonary Angioplasty for Chronic Thromboembolic Pulmonary Hypertension

**DOI:** 10.1161/JAHA.118.008838

**Published:** 2018-06-21

**Authors:** Kenichi Yanaka, Kazuhiko Nakayama, Toshiro Shinke, Yuto Shinkura, Yu Taniguchi, Hiroto Kinutani, Naoki Tamada, Hiroyuki Onishi, Yasunori Tsuboi, Seimi Satomi‐Kobayashi, Hiromasa Otake, Hiroshi Tanaka, Yutaka Okita, Noriaki Emoto, Ken‐ichi Hirata

**Affiliations:** ^1^ Division of Cardiovascular Medicine Department of Internal Medicine Kobe University Graduate School of Medicine Kobe Japan; ^2^ Division of Cardiovascular Surgery Department of Surgery Kobe University Graduate School of Medicine Kobe Japan; ^3^ Department of Clinical Pharmacy Kobe Pharmaceutical University Kobe Japan

**Keywords:** balloon pulmonary angioplasty, chronic thromboembolic pulmonary hypertension, extensive revascularization, pulmonary circulation, pulmonary embolism, pulmonary endarterectomy, pulmonary hypertension, residual, persistent, or recurrent pulmonary hypertension and symptoms, Revascularization, Cardiovascular Surgery, Embolism, Thrombosis, Vascular Disease

## Abstract

**Background:**

Residual symptoms after pulmonary endarterectomy (PEA) remain as the clinical issues to be solved. Additional balloon pulmonary angioplasty (BPA) after PEA showed its efficacy with symptoms in a case series, although long‐term spontaneous recovery of exercise ability after PEA was also reported. However, no studies have validated the clinical efficacy of additional BPA by directly comparing PEA with and without BPA. The aim of this study was to retrospectively evaluate the efficacy of additional BPA as a sequential hybrid therapy for chronic thromboembolic pulmonary hypertension after PEA.

**Methods and Results:**

Among 44 patients with chronic thromboembolic pulmonary hypertension, 20 patients had residual symptoms after PEA. Of those, 10 patients underwent additional BPA (hybrid group) and were compared with the other 10 patients, who were followed up without BPA (PEA group). The period from PEA to additional BPA was 7.3±2.3 months. In hybrid group, mean pulmonary arterial pressure was significantly improved by PEA (40.6±1.8 to 26.9±3.1 mm Hg, *P*=0.001) and improved further (to 16.7±1.8 mm Hg, *P*=0.002) with additional BPA, which resulted in remarkable improvement in World Health Organization (WHO) functional class (pre‐ to post‐BPA: class I/II/III/IV, 0/5/4/1 to 7/3/0/0; *P*<0.001). Compared with the PEA group at follow‐up, the hybrid group achieved better mean pulmonary arterial pressure (18.7±1.7 versus 30.2±3.2 mm Hg, *P*=0.008), WHO functional class (class I/II/III/IV, 7/3/0/0 versus 0/8/2/0; *P*=0.001), and 6‐minute walking distance (429±38 versus 319±22 m, *P*=0.028).

**Conclusions:**

A sequential hybrid strategy improved residual symptoms and exercise capacity compared with single‐PEA therapy.

**Clinical Perspective**

**What Is New?**

- This study is the first direct retrospective comparison of sequential hybrid therapy with single‐pulmonary endarterectomy therapy for chronic thromboembolic pulmonary hypertension.

**What Are the Clinical Implications?**

- Additional balloon pulmonary angioplasty after pulmonary endarterectomy as sequential hybrid therapy for chronic thromboembolic pulmonary hypertension is an effective and safe strategy to improve residual symptoms and exercise capacity.

Chronic thromboembolic pulmonary hypertension (CTEPH) is characterized by stenoses and obstruction of the pulmonary arteries caused by organized thrombus.^1^, ^2^, ^3^, ^4^, ^5^ Without being treated, the prognosis is so poor that a 5‐year survival rate in patients with a mean pulmonary artery pressure (mPAP) >50 mm Hg is 10%.^6^ Pulmonary endarterectomy (PEA) is a gold standard therapy^7^, ^8^; however, PEA for organized thrombus located peripherally in subsegmental pulmonary arteries was less effective and had a high perioperative mortality rate.^7^, ^9^ Presently, balloon pulmonary angioplasty (BPA) can be recommended as an established treatment for nonoperable CTEPH patients.^10^, ^11^, ^12^, ^13^, ^14^, ^15^, ^16^

Residual pulmonary hypertension (PH) after PEA occurs in ≈10% to 35% of patients^4^, ^8^, ^17^, ^18^, ^19^ and is the issue to be solved. It can cause persistent symptoms. The latest treatment algorithm in the European Society of Cardiology guidelines suggests additional BPA as the treatment choice for patients with symptomatic PH after PEA. A previous study reported a promising effect of additional BPA for recurrent PH after PEA.^20^ However, clinical evidence of the therapy for residual PH or symptoms after PEA has not been well accumulated. In addition, the impacts on exercise capacity and pulmonary perfusion of additional BPA are unknown. The purpose of this study was to retrospectively evaluate the efficacy and safety of sequential hybrid therapy with PEA and additional BPA.

## Methods

The data that support the findings of this study are available from the corresponding author on reasonable request.

### Study Design

**Figure 1:**
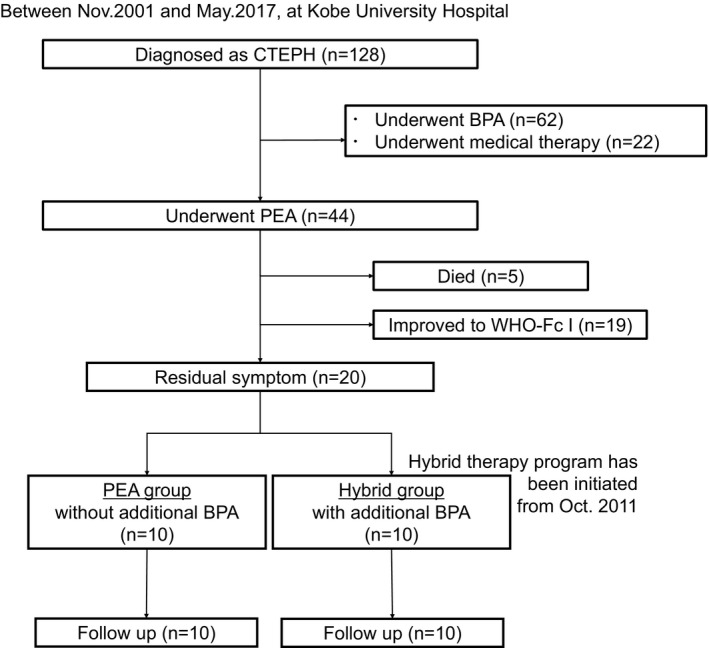
Observational study flow chart. BPA indicates balloon pulmonary; CTEPH, chronic thromboembolic pulmonary hypertension; PEA, pulmonary endarterectomy, WHO‐Fc, World Health Organization functional class.

Between November 2001 and May 2017, 128 patients were diagnosed with CTEPH at Kobe University Hospital. The indication for PEA was determined in conference between cardiologists and cardiovascular surgeons, according to the criteria,^13^, ^21^ in which we considered clot accessibility, age, and comorbidities (Table 1). By this assessment, 44 patients underwent PEA. Among them, 19 patients improved and did not have residual symptoms (World Health Organization functional class I [WHO‐Fc I]), 5 died, and 20 had residual symptom (WHO‐Fc I/II/III/IV: 0/11/9/0) at 6 months after PEA (Figure 1). In 2011, we initiated an additional BPA strategy for the 10 patients (hybrid group) with residual symptoms. The 10 patients in the hybrid group had residual symptoms and residual lesions, maintained activities of daily living, and accepted informed consent. Because we previously reported the clinical utility of extensive revascularization by BPA beyond normalization of hemodynamics,^22^ our inclusion criteria for additional BPA in this study was also determined to be patients with residual symptoms and confirmation of recognized pulmonary lesions for BPA regardless of residual PH. We compared the hybrid group with the other 10 residual‐symptom patients (PEA group) who followed up without additional BPA. All patients provided informed written consent. The design of the present study was approved by the ethics committee of Kobe University (approval no. 160030) and conformed to the tenets of the Declaration of Helsinki.

**Table 1:** Main Reasons of Nonoperability for PEA

Reason	Result
Nonoperable patients	
Patients, n	84
Age, y	66±1.4
BPA, n	62 (73.8)
Medication, n	22 (26.2)
Main reasons for nonoperability, n (%)	
Clot inaccessibility	39 (46.4)
Advanced age^a^	26 (31.0)
Severe COPD	5 (6.0)
Lung cancer	2 (2.4)
Rejection	9 (10.7)
Mild PH^b^	2 (2.4)
Others	1 (1.2)

Values are mean±SEM or n (%).

BPA indicates balloon pulmonary angioplasty; COPD, chronic obstruction pulmonary disease; PEA, pulmonary endarterectomy; PH, pulmonary hypertension.

a Advanced age is defined as >75 y.

b The definition of mild PH is <30 mm Hg of mean pulmonary arterial pressure.

### Analysis of Clinical Parameters

We performed right heart catheterization (RHC) at 5 time points in the hybrid group (pre‐PEA, post‐PEA, pre‐BPA, post‐BPA, follow‐up). RHC at each point was performed at a preoperative period, within 1 month after PEA, just before the first BPA, 1 week after the last BPA, and ≈0.5 year after the last BPA. The data at follow‐up in the PEA group were used from RHC at >1 year after PEA. Pulmonary artery pressure, right atrial pressure, and pulmonary capillary wedge pressure were directly measured, and cardiac output was assessed by the Fick principle with assumed oxygen consumption and calculated pulmonary vascular resistance (PVR). Exercise tolerance was evaluated by 6‐minute walking distance (6MWD) and a cardiopulmonary exercise test. Symptoms were classified by WHO‐Fc. Oxygenation was examined in blood gas analysis at RHC and with pulse oximetry at the cardiopulmonary exercise test. Respiratory function and BNP (brain natriuretic peptide) were also evaluated. Lung ventilation–perfusion scintigraphy was performed again after PEA to assess the residual perfusion defects.

### PEA Procedure

PEA was performed using techniques similar to those established by the San Diego group.^17^, ^23^ Bilateral PEA adopted the median sternotomy under the institution of cardiopulmonary bypass. Distal endarterectomy was conducted with intermittent circulatory arrest for a period limited to 20 minutes with deep hypothermia, which is maintained at 16°C in central temperature.

### BPA Procedure

BPA was performed using techniques similar to those described previously.^12^, ^13^ We placed a short 9‐Fr sheath into the femoral or jugular vein, and then brought the 6‐Fr–long sheath introducer to the main pulmonary artery using a 5‐Fr pigtail catheter. Next, we engaged a target segmental or subsegmental pulmonary artery using a 6‐Fr guide catheter through the long sheath. We performed selective pulmonary angiography to identify any stenoses or occlusions and passed a 0.014‐in guide wire through the target lesions under a support of a microcatheter.

The BPA procedures were repeated until all recognized lesions were treated. Reperfusion pulmonary injury (RPI) was assessed within 4 hours after every BPA by chest computed tomography.

### Statistical Analysis

Quantitative values are presented as mean±SE, and differences in values were tested using the Student *t* test and ANOVA. Nominal data were expressed as numbers and percentages and analyzed using the χ^2^ test. WHO‐Fc was presented as the median and number of patients in each class. Differences in WHO‐Fc were tested by the Mann–Whitney test. *P*<0.1 was considered statistically significant because of the small sample size in the study. All statistical analyses were performed using SPSS Statistics 24.0 (IBM Corp).

## Results

### Clinical Features of the Cases With Residual Symptoms After PEA

The characteristics before and after PEA are shown in Tables 2 and 3. The patients with residual symptoms were of advanced age and had longer disease duration but similar severity with regard to preoperative hemodynamics, WHO‐Fc, 6MWD, and oxygenation parameters compared with patients whose symptoms were fully relieved to WHO‐Fc I (Table 2). Against this, postoperative parameters from the patients with residual symptoms showed significantly worse hemodynamics and lower oxygenation than the WHO‐Fc I group. Although symptoms and 6MWD in both groups were improved from the acute phase (less than one month) to midterm (3 months to one year) after PEA, after PEA, the patients with residual symptoms tended to still have symptoms and reduced exercise capacity even 6 months after PEA (Table 3).

**Table 2:** Basic Characteristics Before PEA

Variable	WHO‐Fc I at 3 Months After PEA	Residual Symptom at 3 Months After PEA	Death Cases	*P* Value (ANOVA)
Basic characteristics				
Patients, n	19	20	5	···
Age, y	51.7±3.6	62.5±2.7^a^	68.7±5.8^a^	0.008
Female	12 (63)	12 (60)	4 (80)	0.824
Body mass index, kg/m^2^	22.0±0.7	22.1±0.8	21.5±1.3	0.939
Disease duration, y	2.9±1.0	7.3±1.1^a^	3.3±1.4	0.011
Proximal lesion type	14 (74)	19 (95)	4 (80)	0.150^b^
WHO‐Fc I/II/III/IV, n	0/4/13/2	0/6/11/3	0/0/2/3	0.176^c^
6MWD, m, mean±SEM (n)	388±12 (8)	326±17 (15)	70 (1)	0.024
BNP, pg/mL	312±16	452±113	1009±251^a^	0.025
RHC				
RAP, mm Hg	9.6±2.9	5.3±1.0	14.8±2.6^d^	0.022
mPAP, mm Hg	44.9±3.3	42.7±1.6	55.4±7.6^d^	0.058
PCWP, mm Hg	12.1±2.4	8.4±1.0	13.0±1.3^d^	0.049
PVR, dyne·s/cm^5^	861±20	922±89	1404±341^a^	0.067
Cardiac index, L/min/m^2^	2.1±0.9	1.9±0.1	1.7±0.3	0.408
Heart rate, beats/min	80±4	71±3^a^	90±10✝	0.031
SaO_2_, %	91.7±2.2	91.2±1.1	92.2±3.3	0.911
SvO_2_, %	61.3±3.3	61.0±1.9	55.7±10.3	0.677
Supportive therapy				
PH monotherapy	7 (37)	9 (45)	2 (40)	0.908^b^
PH combination therapy	3 (16)	3 (15)	1 (20)	1.000^b^
ERA	5 (26)	4 (20)	1 (20)	0.879^b^
PDE5i	0 (0)	0 (0)	1 (20)	0.114^b^
sGCs	3 (16)	7 (35)	1 (20)	0.433^b^
Oral prostacyclin analogue	5 (26)	5 (25)	2 (40)	0.793^b^
Warfarin	19 (100)	20 (100)	5 (100)	1.000^b^
Home oxygen therapy	8 (42)	8 (40)	2 (40)	1.000^b^

Values are mean±SEM or n (%) except as noted. 6MWD indicates 6‐minute walking distance; BNP, brain natriuretic peptide; ERA, endothelin receptor antagonist; mPAP, mean pulmonary arterial pressure; PCWP, pulmonary capillary wedge pressure; PDE5i, phosphodiesterase type 5 inhibitor; PEA, pulmonary endarterectomy; PH, pulmonary hypertension; PVR, pulmonary vascular resistance; RAP, right atrial pressure; RHC, right heart catheterization; SaO_2_, arterial oxygen saturation; sGCs, soluble guanylate cyclase stimulator; SvO_2_, mixed venous oxygen saturation; WHO‐Fc, World Health Organization functional class.

a *P*<0.05 vs WHO‐Fc I group.

b *P* value by χ^2^ test.

c *P* value by Mann–Whitney test.

d *P*<0.05 vs residual symptom group.

**Table 3:** Clinical Parameters After PEA

Variable	WHO‐Fc I (n=19)	Residual Symptoms (n=20)	*P* Value (Unpaired t Test)
Midterm post‐PEA characteristics^a^			
WHO‐Fc I/II/III/IV, n	19/0/0/0	0/14/6/0	<0.001^b^
Period from PEA to 6MWT, mo	6.8±1.7	6.3±1.6	0.914
6MWD, m, mean±SEM (n)	478±10 (3)	334±40 (14)	0.144
Period from PEA to BNP, mo	6.7±2.0	6.5±1.0	0.884
BNP, pg/mL, mean±SEM (n)	57±7 (11)	193±46 (18)	0.078
Acute post‐PEA characteristics^a^			
WHO‐Fc I/II/III/IV, n	5/13/1/0	0/11/8/1	<0.001^b^
Period from PEA to 6MWT, wk	2.8±1.7 (2)	3.8±0.6 (10)	0.469
6MWD, m, mean±SEM (n)	468±13 (2)	260±48 (10)	0.109
Period from PEA to BNP, wk	6.0±4.4	6.4±1.6	0.798
BNP, pg/mL, mean±SEM (n)	126±14 (13)	253±45	0.017
RHC at acute phase after PEA^a^			
Patients, n	19	18	···
Period from PEA, wk	3.5±1.7	2.5±0.3	0.304
Residual PH	4 (21)	8 (44)	0.129^c^
RAP, mm Hg	5.8±0.9	6.1±0.9	0.816
mPAP, mm Hg	19.5±1.4	26.0±2.0	0.011
PCWP, mm Hg	8.9±1.0	9.1±0.9	0.930
PVR, dyne·s/cm^5^	215±26	460±69	0.003
Cardiac index, L/min/m^2^	3.1±0.2	2.3±0.2	0.008
Heart rate, beats/min	85±4	80±4	0.327
SaO_2_, %	95.8±0.7	92.4±1.0	0.011
SvO_2_, %	69.2±1.4	57.9±1.8	<0.001

Values are mean±SEM or n (%) except as noted. 6MWD indicates 6‐minute walking distance; 6MWT, 6‐minute walking test; BNP, brain natriuretic peptide; mPAP, mean pulmonary arterial pressure; PCWP, pulmonary capillary wedge pressure; PEA, pulmonary endarterectomy; PH, pulmonary hypertension; PVR, pulmonary vascular resistance; RAP, right atrial pressure; RHC, right heart catheterization; SaO_2_, arterial oxygen saturation; SvO_2_, mixed venous oxygen saturation; WHO‐Fc, World Health Organization functional class.

a Acute phase is defined as less than one month after PEA. Midterm is defined as 3 months to one year after PEA.

b *P* value by Mann–Whitney test.

c *P* value by χ^2^ test.

### Baseline Comparison Between Study Participants With Residual Post‐PEA Symptoms

We compared post‐PEA characteristics as the baseline for this study comparing the PEA and hybrid groups (Table 4). Almost all parameters including age, disease duration, comorbidity, symptoms, exercise capacity, hemodynamics, residual pulmonary stenosis, and contents of supportive therapy were similar between groups. Only the percentage of female and forced expiratory volume percentage in 1 second were significantly lower in the hybrid group than the PEA group.

**Table 4:** Baseline Comparison Between Study Participants With Residual Post‐PEA Symptoms

Variable	PEA Group (n=10)	Hybrid Group (n=10)	*P* Value (Unpaired *t* Test)
Basic characteristics			
Age, y	61.0±4.8	63.9±2.5	0.593
Female, n	3 (30)	9 (90)	0.006^a^
Disease duration, y	8.1±1.9	6.6±1.2	0.531
Comorbidity			
Lung disease, n	3 (30)	2 (20)	0.606^b^
Left heart disease, n	2 (20)	2 (20)	1.000^b^
Kidney failure, n	1 (10)	0 (0)	0.305^b^
Liver dysfunction, n	0 (0)	1 (10)	0.305^b^
Malignant tumor, n	1 (10)	0 (0)	0.305^b^
Midterm post‐PEA characteristics^c^			
WHO‐Fc I/II/III/IV, n	0/7/3/0	0/5/4/1	0.313^a^
Period from PEA to 6MWT, mo	5.7±0.6	6.7±2.6	0.789
6MWD, m, mean±SEM (n)	325±30 (5)	338±62 (9)	0.881
Period from PEA to BNP, mo	6.2±1.2	6.7±2.2	0.856
BNP, pg/mL, mean±SEM (n)	202±65 (8)	214±67 (10)	0.901
Acute post‐PEA characteristics^c^			
WHO‐Fc I/II/III/IV, n	0/5/5/0	0/6/3/1	0.830^a^
Period from PEA to 6MWT, wk	4.5±1.2	3.4±0.6	0.359
6MWD, m, mean±SEM (n)	270±40 (4)	263±68 (6)	0.937
Period from PEA to BNP, wk	5.2±1.6	7.6±2.4	0.392
BNP, pg/mL, mean±SEM (n)	260±55 (9)	246±73 (10)	0.873
RHC at acute phase after PEA^c^			
Patients, n	8	10	···
Period from PEA, wk	2.8±0.4	2.3±0.4	0.414
Residual PH	4 (50)	4 (40)	0.671^b^
RAP, mm Hg	6.1±0.8	6.1±1.5	0.989
mPAP, mm Hg	24.9±2.5	26.9±3.1	0.633
PCWP, mm Hg	10.5±1.3	7.9±1.2	0.156
PVR, dyne·s/cm^5^	322±62	570±103	0.070
Cardiac index, L/min/m^2^	2.6±0.2	2.0±0.2	0.030
Heart rate, beats/min	79±4	81±6	0.707
SaO_2_, %	93.1±1.8	91.8±1.1	0.544
SvO_2_, %	61.5±2.2	54.8±2.4	0.061
Pulmonary angiography			
Segments with residual lesion, n	8.8±1.0	9.9±1.1	0.467
Lung function, %, mean±SEM (n)			
%VC	71.9±6.9 (6)	83.2±6.5 (8)	0.264
FEV1.0%	78.5±1.5 (6)	70.5±2.3 (8)	0.018
DLCO	56.3±2.0 (4)	60.1±1.9 (8)	0.634
DLCO/VA	55.1±5.0 (5)	53.1±4.5 (8)	0.971
Supportive therapy			
Monotherapy	3 (30)	2 (20)	0.606^b^
Combination therapy	0 (0)	0 (0)	1.000^b^
ERA	0 (0)	1 (10)	0.305^b^
PDE5i	0 (0)	0 (0)	1.000^b^
sGCs	1 (10)	1 (10)	1.000^b^
Oral prostacyclin analogue	2 (20)	0 (0)	0.136^b^
Warfarin	10 (100)	10 (100)	1.000^b^
Home oxygen therapy	3 (30)	5 (50)	0.361^b^

Values are mean±SEM or n (%) except as noted. %VC indicates percentage of vital capacity; 6MWD, 6‐minute walking distance; 6MWT, 6‐minute walking test; BNP, brain natriuretic peptide; DLCO, carbon monoxide diffusing capacity; ERA, endothelin receptor antagonist; FEV1.0%, percentage of forced expiratory volume in 1 s; mPAP, mean pulmonary arterial pressure; PCWP, pulmonary capillary wedge pressure; PDE5i, phosphodiesterase type 5 inhibitor; PEA, pulmonary endarterectomy; PH, pulmonary hypertension; PVR, pulmonary vascular resistance; RAP, right atrial pressure; RHC, right heart catheterization; SaO_2_, arterial oxygen saturation; sGCs, soluble guanylate cyclase stimulator; SvO_2_, mixed venous oxygen saturation; VA, alveolar volume; WHO‐Fc, World Health Organization functional class.

a *P* value by Mann–Whitney test.

b *P* value by χ^2^ test.

c Acute phase is defined as less than one month after PEA. Midterm is defined as 3 months to one year after PEA.

### Procedure and Complications of Additional BPA

The procedure of additional BPA is summarized in Table 5. The period from PEA to BPA was 7.3±2.3 months. The average number of BPA sessions was 2.4±0.3. We mainly dilated lesions in subsegmental arteries that were predominantly distributed in the left lower, right lower, and right upper lobes. The most frequently seen lesion type was web (63.9%), followed by abrupt vascular narrowing (20%). The average balloon size was 3.53±1.31 mm.

**Table 5:** Procedure and Complications of Additional BPA

BPA Procedure and Complications	Result
Patients, n	10
Total sessions, n	24
Period from PEA to BPA, mo	7.3±2.3
Sessions, n	2.4±0.3
Target segments per patient, n	7.8±1.2
Total target vessels, n	155
Balloon size, mm	3.53±1.31
Target distribution of 155 lesions	
Right upper lobe	36 (23.2)
Right middle lobe	10 (6.5)
Right lower lobe	43 (27.7)
Left upper lobe	11 (7.1)
Lingular segment	4 (2.6)
Left lower lobe	51 (32.9)
Target location of 155 lesions	
Lobar artery	0 (0)
Segmental artery	29 (18.7)
Subsegmental artery	126 (81.3)
Target lesion type of 155 lesions	
Web	99 (63.9)
Ring‐like stenosis	12 (7.7)
Abrupt vascular narrowing	31 (20)
Complete vascular obstruction	13 (8.4)
Pouch	0 (0)
BPA complications in 24 sessions	
RPI	8 (33.3)
Hemosputum	3 (12.5)
Only CT findings	5 (20.8)
Wire perforation	2 (8.3)
NPPV	2 (8.3)
Mechanical ventilator	0 (0)
ECMO	0 (0)
Death	0 (0)

Values are mean±SEM or n (%) except as noted. BPA indicates balloon pulmonary angioplasty; CT, computed tomography; ECMO, extracorporeal membrane oxygenation; NPPV, noninvasive positive pressure ventilation; PEA, pulmonary endarterectomy; RPI, reperfusion pulmonary injury.

Complications due to BPA are also shown in Table 5. RPI was the major complication of BPA and occurred in 8 sessions (33.3%). In 3 of 24 sessions, patients produced hemosputum and were supported by noninvasive positive pressure ventilation; however, these patients improved and did not need noninvasive positive pressure ventilation the next day. Five cases of RPI were asymptomatic and found only by computed tomography. No patient died or had severe complications requiring mechanical ventilation or extracorporeal membrane oxygenation after additional BPA.

### Changes in Clinical Parameters With Hybrid Therapy

**Figure 2:**
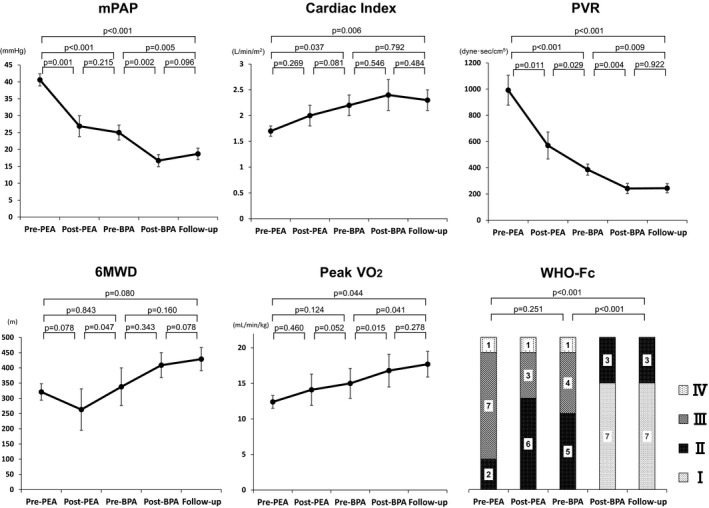
Changes of clinical parameters by hybrid therapy. Each clinical parameter was evaluated at 4 points. Marked improvement of mPAP and PVR by PEA was followed by gradual improvement of cardiac index, 6MWD, and peak VO_2_. Additional BPA for residual pulmonary hypertension and symptoms further improved mPAP, PVR, peak VO_2_, and WHO‐Fc. 6MWD indicates 6‐minute walking distance; BPA, balloon pulmonary angioplasty; mPAP, mean pulmonary arterial pressure; PEA, pulmonary endarterectomy; PVR, pulmonary vascular resistance; VO_2_, oxygen consumption; WHO‐Fc, World Health Organization functional class.

As shown in Figure 2 and Table 6, PEA remarkably improved mPAP and PVR (mPAP: 40.6±1.8 to 26.9±3.1 mm Hg, *P*=0.001; PVR: 992±114 to 570±103 dyne·s/cm^5^, *P*=0.011) together with cardiac index (1.7±0.1 to 2.0±0.2 L/min per m^2^, *P*=0.269). These improvements were also observed during the waiting period from post‐PEA to pre‐BPA. Additional BPA further improved hemodynamics (mPAP: 25.0±2.2 to 16.7±1.8 mm Hg, *P*=0.002; PVR: 386±42 to 242±39 dyne·s/cm^5^, *P*=0.004; cardiac index: 2.2±0.2 to 2.4±0.3 L/min per m^2^, *P*=0. 546), which were maintained through follow‐up. Although 6MWD and minute ventilation–carbon dioxide production slope recovery fell behind peak oxygen consumption improvement in the postoperative period, all indexes of exercise capacity tended to improve following additional BPA (6MWD: 338±62 to 429±38 m, *P*=0.160; peak oxygen consumption: 15.0±2.1 to 17.7±1.8 mL/min per kg, *P*=0.041; minute ventilation–carbon dioxide production slope: 38.9±5.2 to 33.7±2.3, *P*=0.238). Oxygenation at rest was mainly improved by PEA (arterial oxygen saturation at RHC: 90.0±1.5 to 95.6±0.7%, *P*=0.005; arterial oxygen saturation as measured by pulse oximetry at baseline of cardiopulmonary exercise test: 93.6±1.0 to 94.8±0.9%, *P*=0.080). In contrast, oxygenation at exercise was predominantly improved by BPA (minimum arterial oxygen saturation at cardiopulmonary exercise test: 88.0±1.2 to 91.1±1.1%, *P*=0.023). However, oxygenation parameters including arterial oxygen saturation as measured by pulse oximetry and percentage of carbon monoxide diffusing capacity were not normalized even after hybrid therapy. BNP consistently improved with each therapy. Residual symptoms after PEA were dramatically improved by additional BPA (Figure 2, Table 6).

**Table 6:** Time Course of Clinical Parameters Under Sequential Hybrid Therapy

	Pre‐PEA	Post‐PEA	Pre‐BPA	Post‐BPA	Follow‐up	Change by PEA (Pre‐PEA and Pre‐BPA) (*P* Value)	Change by BPA (Pre‐BPA and Follow‐up) (*P* Value)	Change by Hybrid Therapy (Pre‐PEA and Follow‐up) (*P* Value)
Period from PEA, mo	−2.8±0.5	0.5±0.1	7.0±2.3	10.2±2.1	18.2±3.7			
WHO‐Fc I/II/III/IV	0/2/7/1	0/6/3/1	0/5/4/1	7/3/0/0	7/3/0/0	0.251^a^	<0.001^a^	<0.001^a^
BNP, pg/mL	332±99	246±73	214±67	135±51	142±52	0.240	0.017	0.053
RHC								
RAP, mm Hg	3.5±0.8	6.1±1.5	4.3±1.0	3.0±1.2	4.0±1.2	0.269	0.841	0.735
mPAP, mm Hg	40.6±1.8	26.9±3.1	25.0±2.2	16.7±1.8	18.7±1.7	<0.001	0.005	<0.001
PCWP, mm Hg	8.8±1.6	7.9±1.2	9.2±1.1	7.2±1.3	9.1±1.3	0.843	0.952	0.909
Cardiac index, L/min/m^2^	1.7±0.1	2.0±0.2	2.2±0.2	2.4±0.3	2.3±0.2	0.037	0.792	0.006
Heart rate, beats/min	65±3	81±6	80±5	64±4	63.9±1.9	0.071	0.009	0.641
PVR, dyne·s/cm^5^	992±114	570±103	386±42	242±39	244±35	<0.001	0.009	<0.001
SaO_2_, %	90.0±1.5	91.8±1.1	95.6±0.7	94.7±0.7	94.0±0.6	0.005	0.081	0.055
SvO_2_, %	60.1±2.4	54.8±2.4	66.6±2.7	65.1±1.7	65.3±1.5	0.046	0.670	0.075
Exercise capacity, mean±SEM (n)								
6MWD, m	321±27 (9)	263±68 (7)	338±62 (9)	409±41 (7)	429±38 (9)	0.843	0.160	0.080
Peak VO_2_, mL/min/kg	12.4±0.9 (7)	14.1±2.2 (6)	15.0±2.1 (8)	16.8±2.3 (8)	17.7±1.8 (9)	0.124	0.041	0.044
VE/VCO_2_ slope	43.9±2.4 (7)	47.9±6.4 (6)	38.9±5.2 (8)	33.6±2.8 (8)	33.7±2.3 (9)	0.080	0.238	0.024
SpO_2_ at baseline of CPX, %	93.6±1.0 (7)	95.7±0.4 (6)	94.8±0.9 (8)	95.9±0.4 (8)	95.2±0.3 (9)	0.080	0.685	0.111
Minimum SpO_2_ during CPX, %	88.6±0.8 (7)	88.3±1.4 (6)	88.0±1.2 (8)	91.4±1.1 (8)	91.1±1.0 (9)	0.656	0.023	0.093
Lung function								
%VC, %	88.5±4.3	83.2±6.5	86.4±5.3	92.7±3.7	91.5±4.3	0.418	0.134	0.267
FEV1.0%, %	71.3±2.7	70.5±2.3	73.2±1.4	72.2±1.1	74.0±1.1	0.383	0.563	0.200
DLCO, %	68.6±9.2	53.1±4.5	52.2±4.2	56.3±3.7	58.7±3.3	0.073	0.060	0.245
DLCO/VA, %	67.2±3.8	60.1±1.9	57.9±2.9	60.2±3.2	62.4±1.9	0.024	0.185	0.086

Values are mean±SEM or n (%) except as noted. *P* value was calculated by paired *t* test. %VC indicates percentage of vital capacity; 6MWD, 6‐minute walking distance; BNP, brain natriuretic peptide; BPA, balloon pulmonary angioplasty; CPX, cardiopulmonary exercise test; DLCO, carbon monoxide diffusing capacity; FEV1.0%, percentage of forced expiratory volume in 1 s; mPAP, mean pulmonary arterial pressure; PCWP, pulmonary capillary wedge pressure; PEA, pulmonary endarterectomy; PVR, pulmonary vascular resistance; RAP, right atrial pressure; RHC, right heart catheterization; SaO_2_, arterial oxygen saturation; SpO_2_, arterial oxygen saturation as measured by pulse oximetry; SvO_2_, mixed venous oxygen saturation; VA, alveolar volume; VE/VCO_2_, minute ventilation–carbon dioxide production; VO_2_, oxygen consumption; WHO‐Fc, World Health Organization functional class.

a *P* value by Mann–Whitney test.

### Segmental Perfusion Defects Evaluated by Lung Perfusion Scintigraphy

Frequent sites of perfusion defects before PEA were observed in the right middle and lower lobes and the lingular segment of the left lung (Table 7). Despite remarkable improvement of hemodynamics by PEA, residual perfusion defects still remained even after PEA. Additional BPA achieved extensive revascularization in most pulmonary arterial segments.

**Table 7:** Segmental Perfusion Defects Observed by Lung Perfusion Scintigraphy

Variable	Pre‐PEA (n=10)	Post‐PEA (n=10)	Post‐BPA (n=7)
Right A1	6 (60)	5 (50)	1 (14.3)
Right A2	5 (50)	4 (40)	2 (28.6)
Right A3	6 (60)	4 (40)	1 (14.3)
Right A4	8 (80)	5 (50)	0 (0)
Right A5	8 (80)	5 (50)	2 (28.6)
Right A6	5 (50)	3 (30)	1 (14.3)
Right A7	7 (70)	4 (40)	2 (28.6)
Right A8	8 (80)	6 (60)	1 (14.3)
Right A9	7 (70)	4 (40)	1 (14.3)
Right A10	7 (70)	4 (40)	0 (0)
Left A1+2	4 (40)	3 (30)	0 (0)
Left A3	2 (20)	1 (10)	0 (0)
Left A4	7 (70)	7 (70)	4 (57.1)
Left A5	8 (80)	7 (70)	4 (57.1)
Left A6	2 (20)	2 (20)	1 (14.3)
Left A8	5 (50)	5 (50)	1 (14.3)
Left A9	3 (30)	3 (30)	1 (14.3)
Left A10	6 (60)	5 (50)	0 (0)
Number of segments with perfusion defects per a patient, mean±SEM	9.7±0.9	7.4±0.8	2.9±0.9

Although PEA predominately reperfused the right side of lung, residual perfusion defects still remained. Additional BPA achieved extensive revascularization in all segments. Values are n (%) except as noted. BPA indicates balloon pulmonary angioplasty; PEA, pulmonary endarterectomy.

### Comparison of Clinical Parameters Between PEA and Hybrid Groups at Follow‐up

**Figure 3:**
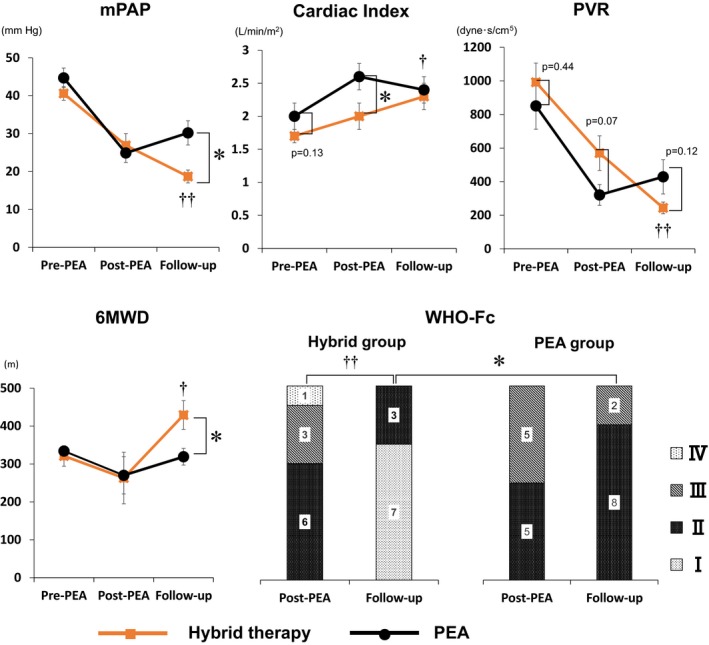
Comparison of changes in clinical parameters between hybrid and PEA groups. Each clinical parameter was compared between groups. *P<0.05 between groups, ^†^ *P*<0.05 vs post‐PEA in the same group. ^††^ *P*<0.01 vs post‐PEA in the same group. 6MWD indicates 6‐minute walking distance; mPAP, mean pulmonary arterial pressure; PEA, pulmonary endarterectomy; PVR, pulmonary vascular resistance; WHO‐Fc, World Health Organization functional class.

When we compared the PEA and hybrid groups, mPAP, symptoms, and exercise capacity in the hybrid group were significantly improved compared with the PEA group (Figure 3, Table 8). During follow‐up, the pulmonary hemodynamics of PEA group worsened slightly (Figure 3). Furthermore, recovery of symptoms and exercise capacity at follow‐up were limited for the PEA group compared with the hybrid group (Figure 3; Table 8), which highlighted the clinical efficacy of additional BPA.

**Table 8:** Clinical Parameters at Follow‐up

Variable	PEA Group (n=10)	Hybrid Group (n=10)	*P* Value (Unpaired *t* Test)
Period from PEA, mo	36.5±17.0	18.2±3.7	0.306
Period from last BPA, mo	NA	8.1±2.2	NA
WHO‐Fc I/II/III/IV	0/8/2/0	7/3/0/0	0.001^a^
BNP, pg/mL	158±44	142±54	0.817
Residual PH	6 (60)	1 (10)	0.029^b^
RHC			
RAP, mm Hg	6.3±1.7	4.0±1.2	0.257
mPAP, mm Hg	30.2±3.2	18.7±1.7	0.008
PCWP, mm Hg	11.2±2.1	9.1±1.3	0.399
Cardiac index, L/min/m^2^	2.4±0.2	2.3±0.2	0.653
Heart rate, beats/min	72.9±3.0	63.9±1.9	0.018
PVR, dyne·s/cm^5^	429±101	244±35	0.115
SaO_2_, %	93.3±1.3	94.0±0.6	0.647
SvO_2_, %	63.5±2.2	65.3±1.5	0.508
Exercise capacity, mean±SEM (n)			
6MWD, m	319±22 (8)	429±38 (9)	0.028
Peak VO_2_, mL/min/kg	15.6±2.4 (5)	17.7±1.8 (9)	0.504
VE/VCO_2_ slope	39.0±2.8 (5)	33.7±2.3 (9)	0.180
SpO_2_ at baseline of CPX, %	95.0±1.3 (5)	95.2±0.3 (9)	0.834
Minimum SpO_2_ at CPX, %	88.0±2.0 (5)	91.1±1.0 (9)	0.132
Lung function, mean±SEM (n)			
%VC, %	81.3±6.5 (8)	91.5±4.3 (10)	0.194
FEV1.0%, %	77.7±3.3 (8)	74.0±1.1 (10)	0.271
DLCO, %	61.1±5.1 (8)	58.7±3.3 (10)	0.634
DLCO/VA, %	62.2±4.8 (8)	61.5±2.3 (10)	0.971
Supportive therapy			
PH monotherapy	2 (20)	3 (30)	0.606^b^
PH combination therapy	1 (10)	0 (0)	0.305^b^
ERA	1 (10)	1 (10)	1.000^b^
PDE5i	0 (0)	0 (0)	1.000^b^
sGCs	2 (20)	2 (20)	1.000^b^
Oral prostacyclin analogue	2 (20)	1 (10)	0.531^b^
Warfarin	10 (100)	10 (100)	1.000^b^
Home oxygen therapy	4 (40)	1 (10)	0.121^b^

Values are mean±SEM or n (%) except as noted. %VC indicates percentage of vital capacity; 6MWD, 6‐minute walking distance; BNP, brain natriuretic peptide; BPA, balloon pulmonary angioplasty; CPX, cardiopulmonary exercise test; DLCO, carbon monoxide diffusing capacity; ERA, endothelin receptor antagonist; FEV1.0%, percentage of forced expiratory volume in 1 s; mPAP, mean pulmonary arterial pressure; NA, not available; PCWP, pulmonary capillary wedge pressure; PDE5i, phosphodiesterase type 5 inhibitor; PEA, pulmonary endarterectomy; PH, pulmonary hypertension; PVR, pulmonary vascular resistance; RAP, right atrial pressure; RHC, right heart catheterization; SaO_2_, arterial oxygen saturation; sGCs, soluble guanylate cyclase stimulator; SpO_2_, arterial oxygen saturation as measured by pulse oximetry; SvO_2_, mixed venous oxygen saturation; VA, alveolar volume; VE/VCO_2_, minute ventilation–carbon dioxide production; VO_2_, oxygen consumption; WHO‐Fc, World Health Organization functional class.

a *P* value by Mann–Whitney test.

b *P* value by χ^2^ test.

**Figure 4:**
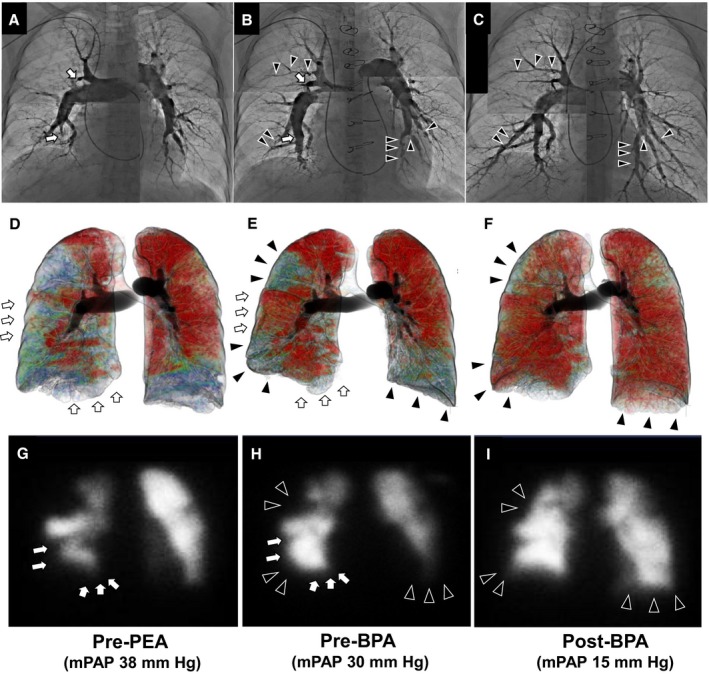
A representative case of hybrid therapy by additional BPA after PEA. Representative pulmonary angiography (A–C), dual‐energy CT (D–F), and lung ventilation–perfusion scintigraphy (G–I). PEA released the complete obstruction in the right anterior branch (right A3) and posterior basal branch (right A10), resulting in peripheral perfusion recovery in the area (white arrow). Four additional BPA sessions in the right peripheral lower lobe (right A8), upper lobe (right A3), and left lower lobe (left A8, A9, A10) improved the residual perfusion defects (black arrow). BPA indicates balloon pulmonary angioplasty; mPAP, mean pulmonary arterial pressure; PEA, pulmonary endarterectomy.

### A Representative Case of Hybrid Therapy

A 61‐year‐old woman (WHO‐Fc III) with a central type of CTEPH showed multiple perfusion defects (Figure 4D and 4G), PH hemodynamics (mPAP: 38 mm Hg; PVR: 858 dyne·s/cm^5^; cardiac index: 2.05 L/min per m^2^ by RHC), and stenoses and obstruction in right A3, A1, A10, A8, and several branches (left A8–10; Figure 4A). Even though PEA improved hemodynamics (mPAP: 30 mm Hg; PVR: 463 dyne·s/cm^5^; cardiac index: 2.16 L/min per m^2^) and released the stenoses in right A3 and A10 (Figure 4B), this patient still had residual PH, symptom (WHO‐Fc II), peripheral lesions, and perfusion defects (Figure 4E and 4H). Four additional BPA sessions improved the residual lesions and perfusion in right A3, A8, and left A8 to A10 (Figure 4C, 4F, and 4I) and normalized her hemodynamics (mPAP: 15 mm Hg; PVR: 193 dyne·s/cm^5^; cardiac index: 3.02 L/min per m^2^) and symptom (WHO‐Fc I).

## Discussion

Residual symptoms after PEA remain an issue to be solved, although previous studies have shown better prognosis for patients who achieved mPAP levels <30 mm Hg by PEA.^24^ We performed additional BPA on 10 patients who had residual symptom after PEA. In this study, hybrid therapy with PEA and additional BPA significantly reduced mPAP compared with single‐PEA therapy and resulted in better exercise capacity (6MWD) and relieved symptoms.

### Residual PH and Symptoms After PEA

Increasing evidence shows incidence of residual PH after PEA ranging from 10.1%^17^ to ≈35%^4^ despite various definitions. In this study, 12 of 39 PEA survivors (30.8%) had residual PH (mPAP ≥25 mm Hg) several weeks after PEA, which is a frequency similar to previous reports. Residual symptom (WHO‐Fc II or higher) is more frequently reported as 69.8% and 62.1% at 3 and 12 months, respectively, after PEA.^24^ Age, preoperative PVR, New York Heart Association class, right atrial pressure, and female sex were reportedly identified as risk factors for residual PH. In this study, we proved that advanced age, longer disease duration (Table 2), postoperative hemodynamics, oxygenation, exercise capacity, and BNP levels (Table 3) were significant risk factors for residual symptoms. In addition, we showed no relationship between severity of preoperative hemodynamics and residual symptom (Table 2). Because only 44% of patients with residual symptom had residual PH (Table 3), it should be noted that residual symptoms occurred with higher frequency compared with residual PH. All patients included in the hybrid group had segmental perfusion defects (Table 7). As such, we advocate advanced monitoring for lung perfusion and treatment for extensive revascularization to manage residual symptoms.

### Suitable Timing of Additional BPA

The appropriate suitable timing for additional BPA after PEA is unknown. Varied timing of hybrid therapies has already been reported. A previous study showed that additional BPA performed 4.1 years after PEA, in contrast to 7.3 months in our study, could improve hemodynamics of patients with residual or recurrent PH.^20^ Other case series reported 3 CTEPH patients with 1 operable and 1 inoperable side who underwent preoperatively scheduled combined PEA and BPA simultaneously.^25^

A previous study showed prolonged improvement of exercise capacity observed over 1 to 2 years, when whole PEA cases were analyzed.^26^, ^27^ However, when only residual PH cases were analyzed, 6MWD and symptoms improved until 3 months after PEA, with no further improvement afterward.^24^ Many studies have shown that residual PH affects prognosis. Moreover, hemodynamic assessment 3 to 6 months after PEA is important to stratify patients at higher risk of dying of CTEPH.^28^ Consequently, when patients have residual symptoms, we believe it is ideal to determine the indication for sequential hybrid therapy several months after PEA if we find residual pulmonary arterial stenosis by pulmonary angiography. However, further investigation is necessary to determine suitable timing for additional BPA after PEA.

### Mechanism of Efficacy and Goal of Hybrid Strategy

The underlying pathophysiology of residual PH was investigated recently. A suggested mechanism is pulmonary arteriopathy. Jujo et al proved the correlation between hypoxemia and severity of obstruction in the level of arterial resistance.^29^ In contrast, our BPA targets mainly subsegmental arteries (Table 5). Our strategy for BPA was to treat all recognized lesions on pulmonary angiography as shown in our previous report of extensive revascularization by BPA.^22^ This strategy efficiently improved pulmonary perfusion, achieving significantly better hemodynamics and exercise capacity. Even though both residual lesions in subsegmental arteries and arteriopathy in small vessels could cause residual PH, it is important to perform extensive revascularization for residual lesions beyond normalization of hemodynamics as the ideal goal of hybrid therapy.^22^ Our hybrid strategy highlights the definite efficacy of lung perfusion recovery (Table 7) by treating subsegmental lesions on symptom and exercise capacity. Concurrently, persistent hypoxemia and limited percentage of carbon monoxide diffusing capacity even after extensive reperfusion might suggest the limitation of this strategy for underlying arteriopathy or intrapulmonary shunt.^30^ Besides hemodynamic evaluation by RHC, the evaluation of lung perfusion by scintigraphy or dual‐energy computed tomography will be optimal for monitoring.

### Complementary Roles of PEA and BPA

PEA has an advantage for lesions in lobular and segmental arteries^17^ in contrast to subsegmental arteries as favorable BPA targets (Table 5). Although our previous report targeting inoperable CTEPH showed that the average balloon size used was 4.35±1.78 mm,^13^ we used smaller balloons (3.53±1.31 mm) in these 10 cases, suggesting that most residual lesions were located at more peripheral sites. Of note, pouch and complete vascular obstruction were not major targets for these BPA sessions (Table 5) but are more favorable targets for PEA. The surgical indication should be determined by assessing the advantages of both therapies. Furthermore, hybrid therapy may be an ideal strategy that can correspond to diversified lesions and complement the disadvantages of both therapies.

### Safety of Hybrid Therapy

RPI is known as a major complication of BPA. We previously reported that RPI occurred in 64.0% of sessions for inoperable CTEPH, although 50.9% of the RPIs were asymptomatic.^13^ RPI in the present study occurred less frequently, in 33.3% of sessions. Higher pulmonary arterial pressure (mPAP >35 mm Hg) is known as a risk factor for RPI.^12^ Low RPI incidence in this study might be due to low mPAP achieved by prior PEA. The safety of BPA in hybrid therapy is an advantage promoting repeatable procedures.

### Limitations

This study is a retrospective single‐center observational study, and the number of patients, especially those who underwent PEA, was small. Clinically relevant results that may not be statistically significant may be due to the small sample size. The classification into hybrid or PEA group was not allocated randomly but retrospectively. A multicenter prospective randomized study is required for further valuation to determine the role of this strategy.

The long‐term efficacy of sequential hybrid therapy is still unknown and has not been reported for BPA itself. Long‐term analysis is needed to evaluate the true efficacy of BPA in addition to PEA.

Less experience with PEA (44 cases in 17 years) at our center compared with that in Europe and the United States can be a cause of higher mortality (5/44, 11.4%).^31^ We excluded patients >75 years from the indication for PEA because the BPA program was initiated at our institution based on its lower mortality rate after BPA.^13^ The long‐term follow‐up data for the PEA group in this study might be different from results at centers with larger volume of surgery. Our conclusions should be verified at high volume centers.

## Conclusion

Additional BPA after PEA as sequential hybrid therapy for CTEPH is an effective and safe strategy to improve residual symptoms and exercise capacity.

## Disclosures

Kazuhiko Nakayama received research grants from Actelion Pharmaceuticals Japan Ltd, Bayer Holding Ltd, and GlaxoSmithKline plc. Ken‐ichi Hirata received research grants from Actelion Pharmaceuticals Japan Ltd, and Bayer Holding Ltd. The remaining authors have no disclosures to declare.
